# Systematic Humanization of the Yeast Cytoskeleton Discerns Functionally Replaceable from Divergent Human Genes

**DOI:** 10.1534/genetics.120.303378

**Published:** 2020-06-10

**Authors:** Riddhiman K. Garge, Jon M. Laurent, Aashiq H. Kachroo, Edward M. Marcotte

**Affiliations:** * Center for Systems and Synthetic Biology, Department of Molecular Biosciences, The University of Texas at Austin, Texas 78712; † Institute for Systems Genetics, Department of Biochemistry and Molecular Pharmacology, NYU Langone Health, New York 10016; ‡ The Department of Biology, Centre for Applied Synthetic Biology, Concordia University, Montreal, H4B 1R6 Quebec, Canada

**Keywords:** humanized yeast, evolution, gene families, orthologs, cytoskeleton

## Abstract

Many gene families have been expanded by gene duplications along the human lineage, relative to ancestral opisthokonts, but the extent to which the duplicated genes function similarly is understudied. Here, we focused on structural cytoskeletal genes involved in critical cellular processes, including chromosome segregation, macromolecular transport, and cell shape maintenance. To determine functional redundancy and divergence of duplicated human genes, we systematically humanized the yeast actin, myosin, tubulin, and septin genes, testing ∼81% of human cytoskeletal genes across seven gene families for their ability to complement a growth defect induced by inactivation or deletion of the corresponding yeast ortholog. In five of seven families—all but α-tubulin and light myosin, we found at least one human gene capable of complementing loss of the yeast gene. Despite rescuing growth defects, we observed differential abilities of human genes to rescue cell morphology, meiosis, and mating defects. By comparing phenotypes of humanized strains with deletion phenotypes of their interaction partners, we identify instances of human genes in the actin and septin families capable of carrying out essential functions, but failing to fully complement the cytoskeletal roles of their yeast orthologs, thus leading to abnormal cell morphologies. Overall, we show that duplicated human cytoskeletal genes appear to have diverged such that only a few human genes within each family are capable of replacing the essential roles of their yeast orthologs. The resulting yeast strains with humanized cytoskeletal components now provide surrogate platforms to characterize human genes in simplified eukaryotic contexts.

## Abstract (precis)

To understand the extent of functional divergence across duplicated genes in core eukaryotic systems, Garge *et al.* systematically tested ∼81% of human orthologs....

GENE duplication is regarded as one of the key drivers of evolution, contributing to the generation and accumulation of new genetic material within species (Soukup 1970; Koonin 2005). Duplication creates an initial multiplication of dosage and functional redundancy, but the trends dictating how duplicated genes retain function or diverge are still unclear. The processes governing the distribution of molecular roles within gene families also have important consequences for annotating genes, which generally takes advantage of sequence similarity and conservation over vast timescales of divergence to infer functions of homologous genes across species. Many individual studies have directly tested the conservation of function among orthologs from different species by swapping them from one species into another (Heinicke *et al.* 2007; Cherry *et al.* 2012). However, only recently have efforts been made to test such functional equivalence more systematically, with several recent large-scale studies harnessing “the awesome power of yeast genetics” to systematically replace yeast genes by their human, plant, or even bacterial counterparts and assay for functional compatibility (Kachroo *et al.* 2015, 2017; Hamza *et al.* 2015; Laurent *et al.* 2016; Sun *et al.* 2016; Yang *et al.* 2017). Although humans and yeast last shared a common ancestor nearly a billion years ago, these studies have demonstrated that substantial fractions (12%–47%) of tested essential yeast genes could be replaced by their human equivalents (Kachroo *et al.* 2015, 2017; Hamza *et al.* 2015; Sun *et al.* 2016; Yang *et al.* 2017; Laurent *et al.* 2019). The ability of many human genes to functionally replace their yeast orthologs demonstrates the high degree of functional conservation in eukaryotic systems over billion-year evolutionary timescales (Kachroo *et al.* 2015; Laurent *et al.* 2016).

Previous humanization efforts in yeast have focused primarily on ortholog pairs with no obvious duplications within yeast and human lineages (1:1 orthologs), only partially testing the orthologs in expanded gene families (Kachroo *et al.* 2015; Hamza *et al.* 2015; Sun *et al.* 2016; Yang *et al.* 2017; Laurent *et al.* 2019) and seldom beyond assaying impact on growth rate. In this study, to better understand functional conservation across expanded gene families in core eukaryotic processes, we focused on the major structural components of the eukaryotic cytoskeleton, including actins, myosins, septins, and tubulin genes. Genes constituting the eukaryotic cytoskeleton play key roles in critical cellular processes, mainly organizing the contents of the cell by dynamically controlling cell shape, positioning organelles, and transporting macromolecules including chromosomes across the cell through the generation of coordinated mechanical forces (Wickstead and Gull 2007, 2011; Wickstead *et al.* 2010). Importantly, cytoskeletal gene families have undergone large expansions along the human lineage, while being restricted to only a few family members in yeast (Figure 1).

**Figure 1:**
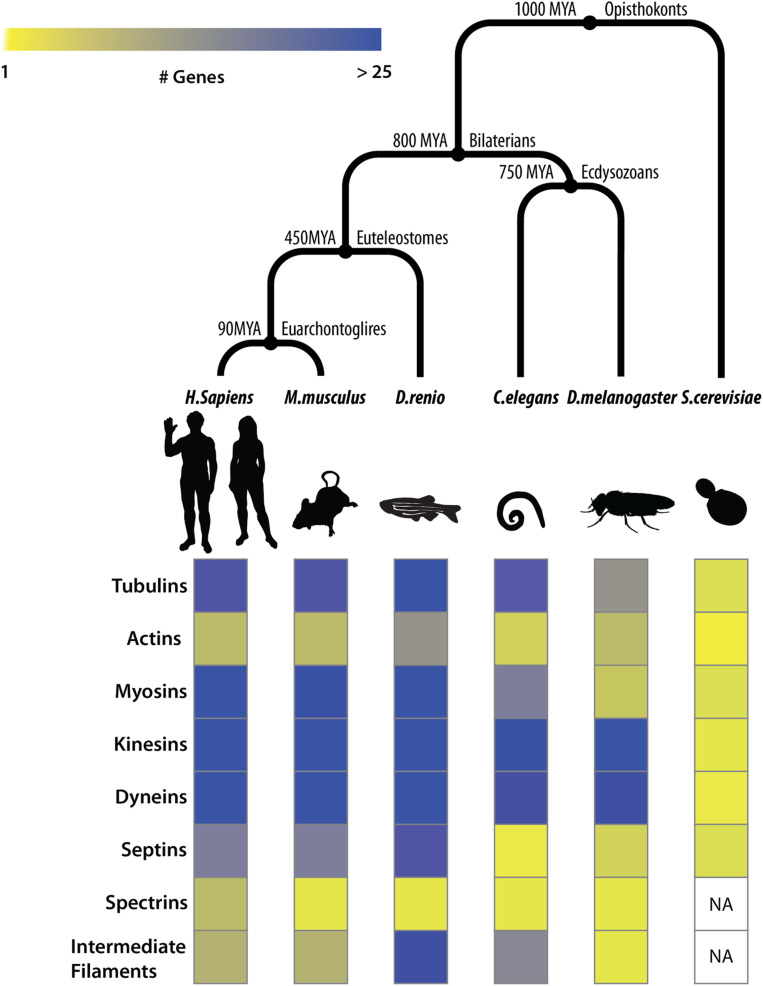
Orthologs in cytoskeletal gene families have undergone extensive duplications in Bilaterians. (Top) Species divergence across opisthokonts. (Bottom) Heatmap depicting the number of orthologs in eukaryotic cytoskeleton gene families (rows) across species (columns). Cytoskeletal ortholog counts for model organisms curated from The Alliance of Genome Resources database (Agapite *et al.* 2020).

Advances in comparative genomics have shed light on the likely cytoskeletal components present in the last eukaryotic common ancestor (LECA) (Wickstead and Gull 2007, 2011; Wickstead *et al.* 2010). Additionally, phylogenetic profiling studies of the cytoskeleton have been highly informative in inferring gene loss and retention events across eukaryotic clades (Wickstead and Gull 2007; Wickstead *et al.* 2010) (Figure 1). Such studies suggest that the origins of the eukaryotic cytoskeleton predate eukaryogenesis and ancestrally trace back to primitive tubulin- and actin-like homologs in bacteria^13^. These components, critical in cell division, subsequently evolved to incorporate families of accessory motors and regulatory proteins expanding toward performing vital cellular roles, including phagocytosis, motility, and vesicular transport, still evident across vast eukaryotic clades of life (Hall and Russell 2004; McKean *et al.* 2001; Wickstead and Gull 2007; Janke 2014; Jékely 2014).

Though the cellular roles of human cytoskeletal gene families have been broadly elucidated, aided by studies in simpler eukaryotes (Schatz *et al.* 1986; Winsor and Schiebel 1997; Bode *et al.* 2003; Nogales *et al.* 2010; Li *et al.* 2011), the specific functions of their constituent family members in humans have still only been partially characterized to date (Tischfield *et al.* 2010; Feng *et al.* 2016; Stottmann *et al.* 2016; Wawro *et al.* 2017; Huang *et al.* 2018; Yuan *et al.* 2018; Chen *et al.* 2019). Functional assays in human cell lines pose the challenge of functional redundancy, with buffering by other paralogs complicating the determination of paralog-specific roles within cytoskeletal gene families. The high degree of sequence conservation among paralogs within each cytoskeletal family make functional analysis of individual cytoskeletal genes directly in human cells both experimentally and computationally cumbersome. However, cross-species gene swaps have the potential to provide direct assays of individual paralogs within these expanded gene families, thereby revealing the extent to which present-day orthologous genes retain ancestral function.

To understand the extent to which human cytoskeletal genes in expanded orthogroups retain cross-species functional equivalence, we systematically humanized major elements of the yeast cytoskeleton. We tested ∼81% (50/62) of all human genes from actin, myosin, septin, and tubulin families, using a combination of classical yeast genetics and CRISPR-Cas9 mediated genome editing to assay the replaceability of essential cytoskeletal orthologs, as initially determined via simple growth rescue complementation assays. Overall, we show that (13/50) members from five of seven tested gene families (actin, heavy myosin, septin, β- and γ-tubulin) can indeed execute essential roles of their yeast counterparts. Within each replaceable family, we show that several present-day human orthologs still possess functional roles of their respective opisthokont ancestors compatible in a yeast cellular context. Additionally, we characterized cellular phenotypes beyond growth and observed differential abilities among complementing human cytoskeletal genes to carry out nonessential cytoskeletal roles, including those in cell morphology, sporulation, mating, meiosis, and cytokinesis. Besides revealing human cytoskeletal orthologs capable of performing their core eukaryotic roles, yeast strains with human cytoskeleton components additionally serve as cellular reagents to study the specific roles of expanded cytoskeletal gene family members in a simplified unicellular eukaryotic context.

## Materials and Methods

### Curating human orthologs to yeast genes

Yeast genes to be tested for replaceability were curated from *Saccharomyces* Genome Database (Cherry *et al.* 1998, 2012), and human orthologs were curated from the InParanoid (Sonnhammer and Östlund 2015) and EggNOG (Huerta-Cepas *et al.* 2016) databases, both of which employ graph-based algorithms that recognize orthogroups between species by exhaustively performing an all-vs-all bidirectional BLAST search of all protein sequences and Hidden Markov Models to estimate ortholog groups, respectively. We curated a total of 62 human cytoskeletal proteins with yeast orthologs in the seven major cytoskeletal gene families (Supplemental Material, Table S2).

### Cloning human cytoskeletal ORFs

Human genes were extracted from the ORFeome (Lamesch *et al.* 2007), a collection of *Escherichia coli* strains, each containing a single human ORF cDNA cloned into a Gateway “entry” vector (Alberti *et al.* 2007). Human genes cloned in this manner are flanked by attL recombination sites. To generate yeast expression vectors, the human entry vectors were isolated and subjected to Gateway LR reactions with a yeast Gateway “destination” vectors followed by transformation into competent *E. coli* to obtain yeast expression clones. We used destination vectors from the Advanced Yeast Gateway kit (Alberti *et al.* 2007), specifically pAG416-GPD-ccdB, pAG416-GPD-ccdB-eGFP, pAG426-GPD-ccdB-eGFP, pAG426-GPD-ccdB (CEN, Ura) destination vectors. Since the original version of pAG416-GPD-ccdB does not encode a stop codon immediately after the cloning region (which is also not encoded in ORFeome genes), this results in a ∼60 amino acid tail being translated to any protein expressed from it. To eliminate potential issues from the tail, we mutagenized the vector downstream of the cloning region to introduce a stop codon, thereby shortening the tail to six amino acids (pAG416-GPD-ccdB+6Stop) (Kachroo *et al.* 2015). Prior to performing complementation assays, all expression clones were verified by Sanger sequencing to ensure there were no sequence errors or mutations in the human cytoskeletal genes prior to complementation assays in yeast.

### Assaying human cross-species complementation in yeast

#### Tetrad dissection and analysis:

In all, 40 human genes were assayed using tetrad dissection (Figure S2A): the yeast heterozygous diploid deletion strain (Magic Marker) collection (Giaever *et al.* 2002) (obtained from ATCC) with one allele replaced with a kanamycin-resistance (KanMX) cassette was used. Human genes in Gateway entry clones (pDON223) were curated from the Human ORFeome collection(Lamesch *et al.* 2007) and cloned into yeast destination vectors. We transformed each human clone or an empty vector (pAG416-GPD-ccdB+6Stop lacking the human ORF) control into the appropriate heterozygous diploid deletion strain yeast strainm and grew them on SC–Ura + G418 (200 µg/ml) to select for the human clone (Ura) and KanMX (G418) simultaneously. Transformants were then plated on GNA-rich presporulation medium containing G418 (200 μg/ml). Individual colonies were inoculated into a liquid sporulation medium containing 0.1% potassium acetate, 0.005% zinc acetate, and were incubated at vigorous shaking at 25° for 3–5 days. Following this, sporulation efficiency was estimated by microscopy, and successful sporulations were subjected to tetrad dissection and analysis using standard protocols (Dunham *et al.* 2015). We initially plate the spores from tetrads on yeast extract peptone dextrose (YPD) medium. This allows us to check for the spore viability. The colonies are then replica-plated onto either YPD+G418, SC-Ura ± G418 or 5-fluorootic acid (5-FOA) media. We predominantly observe two spores with Ura selection that tend to be G418 resistant as well. This behavior could be due to various patterns of segregation of CEN vectors, or that the YPD selection allows the Ura to be lost from the wild-type spores. Successful dissections were replica plated both on 5-FOA (for plasmid counter-selection) and YPD + G418 (for yeast null allele selection). Successful complementations consisted of 2:2 segregation with survival on YPD+G418 and failure to grow on 5-FOA (Figure S2B). We subsequently performed quantitative growth assays (in triplicate) on the tetrads passing the 5-FOA and G418 segregation test (Figure S2, B and C). Each profile (Figure S2C) was analyzed and quantified to detect any growth defects in yeast. Out of 40 human genes assayed in this manner, 10 functionally rescued yeast from the lethal phenotype. (Table S2). For population-based plasmid complementation assays, we selected the spores on SC-Arg-His-Leu+Can-Ura +/− G418. A complementation consists of spores being able to proliferate both in the presence (+G418, selecting for spores with the null allele) and absence (–G418, selecting for wild-type spores) of G418. Assays where this threshold was not met were deemed noncomplementing.

#### Yeast temperature-sensitive assays:

Where available, temperature-sensitive (ts) strains from Li *et al.* (2011) were used to assay human gene complementation. These strains grow ideally at lower permissive (22–26°) but not restrictive (35–37°) temperatures. We transformed each human gene expression vector and empty vector control plasmids (pAG416-GPD-ccdB+6Stop) into the corresponding ts strains (Figure S3A). Transformants were plated on SC-Ura medium at both permissive and restrictive temperatures, allowing us to control for transformation efficiency and expression toxicity while simultaneously testing for functional replacement by the human gene (Figure S3B). Successful complementations consisted of transformants with the human gene surviving under both conditions (growth at permissive or restrictive temperatures), while those with the empty vector failed to survive at restrictive temperatures. Successfully humanized strains were then subjected to quantitative liquid growth assays to monitor robustness of complementation (Figure S3C).

#### Genomic replacement via CRISPR-Cas9:

A total of 29 human genes were assayed using a CRISPR-Cas9 mediated yeast genome editing protocol as previously described (Kachroo *et al.* 2017; Akhmetov *et al.* 2018). For every yeast gene to be replaced endogenously, a minimum of two synthetic guide RNAs (sgRNA) with high on-target and high off-target scores were designed using the Geneious v10.2.6 CRISPR-Cas9 tools suite. Selected sgRNAs were ordered as oligos from IDT and cloned into yeast CRISPR-Cas9 knockout plasmids from the yeast toolkit (YTK) (Lee *et al.* 2015) to express a synthetic guide RNA, Cas9 nuclease, and a selectable marker (Ura) (see Table S4 for guide sequences and primers). Wild-type yeast strains (s288c or BY4741) were transformed with a knockout plasmid and a repair template in the form of a PCR amplicon composed of the human open reading frame flanked with sequence homology to the targeted yeast locus (Figure S4A). Transformants were selected on SC-Ura medium to select for the knockout plasmid. While CRISPR-mediated double-stranded breaks are intrinsically lethal, targeted editing of an essential gene locus acts as an added layer of selection in replacing the human gene of interest, allowing cells to survive only if the human ortholog being assayed functionally replaces the yeast gene at the appropriate locus (Kachroo *et al.* 2017; Akhmetov *et al.* 2018). Surviving colonies (Figure S4B) obtained in the presence of repair templates were screened for successful humanization by colony PCR using primers outside the region of homology. Confirmed clones were Sanger sequenced and subsequently subjected to quantitative liquid growth assays to evaluate overall fitness. In the case of tubulins, all CRISPR-Cas9 assays were carried out in a BY4741 *tub3**Δ* strain to avoid homologous repair of the *TUB1* locus by the *TUB3* gene. To generate diploid strains homozygous for human β-tubulin alleles, we replaced the yeast *TUB2* allele in our heterozygous diploids by retransforming the CRISPR-Cas9 and sgRNA expression vector, specifically targeting yeast β-tubulin allele (*TUB2*), and selecting for viable strains without supplying an external repair template, forcing homology-directed repair of the *TUB2* lesion by the human ortholog(s) on the other homologous chromosome.

### Growth assays

Liquid growth assays were performed in triplicate using a Biotek Synergy HT incubating spectrophotometer in 96-well format. All humanized strains were precultured to saturation in appropriate media, and diluted into 150 µl of medium to finally have 0.05–0.1 × 10^7^ cells/ml. Each growth assay lasted for 48 hr, with absorbance measured at 600 nm every 15 min. For human genes assayed using heterozygous diploid deletion collections, growth assays were performed in YPD, SC-Ura, and YPD+G418 medium to confirm retention of the plasmid and selection of the deletion allele, respectively. For CRISPR assays, humanized strains were grown in YPD. Human genes assayed via ts alleles were grown at both temperatures (permissive and restrictive). Growth curve data were processed in Rstudio and plotted using the ggplot2 package or GraphPad Prism.

### Microscopy and image analysis

For imaging, for plasmid-based complementation assays, all the yeast strains were grown in selection conditions to maintain the plasmid (Ura+). For the genomically integrated humanized yeast strains, the cells were grown in YPD medium.

For 4′,6-diamidino-2-phenylindole (DAPI) staining, cells were grown in liquid medium to saturation and fixed in a final concentration of 3.7% (v/v) formaldehyde for 10 min at room temperature. Cells were then washed with water and 1 M sorbitol, after which they were resuspended in sorbitol (1x of the original volume) and fixed in 50% ethanol (final concentration). The cells were washed and resuspended in 1 M sorbitol. DAPI was then added to the solution to a final concentration of 1 µg/ml and incubated for 5–10 min at room temperature.

Cells were imaged using a Nikon TE-2000-E inverted microscope with an Apo 40x/NA 0.95 objective and Cascade II 512 camera (Photometrics), Lambda LS Xenon light source and Lambda 10-3 filter wheel control (Sutter Instrument) with a motorized stage (Prior Scientific). All imaging and parameters were set via the Nikon NIS Elements Imaging Software. Images were captured at one frame per second through a 89000ET filter set (Chroma Technology) with channels “DIC L”, “FITC” (Ex 490/20, Em 525/36). Green fluorescent protein (GFP) fluorescence images were collected with an exposure time of 1s. DAPI images for the humanized septin yeast strains were obtained using a Zeiss LSM 710 confocal microscope with a Plan-Apochromat 63x/1.4 oil-immersion objective with standard DAPI (Ex 350/50, Em 455/50) wavelength setting and operated using Zeiss ZEN Microscope software.

Cell size measurements were performed with a minimum of 10 fields of view and 2000 cells per strain. Image analysis and quantification was performed using FIJI/ImageJ (Schindelin *et al.* 2012, 2015). For quantifying cell size, edge detection scripts were written as Python scripts and FIJI macros.

### Mating assays

Since the genes assayed via CRISPR were tested in BY4741 (genotype MATa *his3**Δ1 **leu2**Δ0 **met15**Δ0 **ura3**Δ0*), the humanized strains were mated to a BY4742 derivative (genotype MATα *his3**Δ1 **leu2**Δ0 **lys2**Δ0 **ura3**Δ0 **mkt1*-D30G *rme1*-ins308A tao3-E1493Q) so that diploids could be selected on SC-Lys-Met medium. Strains were first patched on a YPD plate. The humanized strain being assayed and its complementary mating strain were streaked perpendicular to each other and were grown overnight. These were then replica plated on SC-LYS-MET medium to select for diploids. In cases of humanized septin mating assays, haploids were mated on YPD as described above and streaked on SC-Lys-Met to select for diploids, following which they were incubated for 3 days before imaging (Figure 5B and Figure S7B). For sporulation, diploids were grown overnight on GNA-rich presporulation medium. Individual colonies from this plate were inoculated into a liquid sporulation medium containing 0.1% potassium acetate, 0.005% zinc acetate, and were incubated at vigorous shaking at 25° for 3–5 days. Spores were dissected on a YPD plate using a similar protocol, as described previously. Dissected spores were replica plated on SC-Lys and SC-Met media to select haploid spores. This assay enabled subsequent mating of humanized haploids to each other, and also generated homozygous diploids for the human genes via CRISPR-Cas9. All heterozygous diploids were scored for spore viability and 2:2 segregation of LYS, MET, and MAT loci.

### Gene tree construction

Maximum likelihood trees for each cytoskeletal family were constructed with the Gamma LG protein model with the rapid hill-climbing algorithm bootstrapping 1000 replicates on human and yeast protein sequences curated from the Uniprot database. All trees were computed using Geneious v10.2.6’s RAxML’s plugin (Stamatakis 2014) (v8.2.11).

### Human cytoskeleton RNA expression analysis

RNA expression profiles across human tissue types were obtained from the Human Protein Atlas (Uhlén *et al.* 2015). We extracted the human cytoskeletal gene set from the available expression data normalized via FPKM. The normalized data were then processed in Rstudio and plotted with ggplot2 using the ggridges package.

### SCMD-SGD database searching and evaluations

Genetic interaction data for each assayed yeast cytoskeletal gene was downloaded from the *Saccharomyces* Genome Database (Cherry *et al.* 1998, 2012) (SGD) to curate all the interaction partners for the assayed yeast cytoskeletal gene set. Imaging data and morphology parameter files were downloaded from the *Saccharomyces* Morphological Database (Saito *et al.* 2004) (SCMD) website. As relevant parameters to the phenotypes observed in the case of actin and septin interactors, we specifically examined the long (C103) and short axis (C104) lengths of mother cells budded (SCMD cell types B and C) and unbudded cells (SCMD cell type A). C103/C104 ratios were calculated and ranked in ascending order. We reasoned that deletion strains resulting in small cells with round phenotypes (similar to our humanized actin yeast strains) would have ratios close to 1. In contrast, abnormally shaped cells (similar to our humanized septin yeast strains) would have larger (>1) C103/C104 ratios. We ranked the deletion collection phenotypes by the long-to-short axis ratios of the budded cells as a measure of circularity. Significance was determined by the hypergeometric test (Table S4).

### Data availability

The authors state that all data necessary for confirming the conclusions presented in the article are represented fully within the article. All strains and plasmids created are available upon request. Table S1 contains all strains used in this study. Table S4 contains all primers used in this study. Supplemental material available at figshare: https://doi.org/10.25386/genetics.12456206.

## Results

### Human cytoskeletal genes can functionally replace their corresponding yeast orthologs

Of the 324 cytoskeleton genes in yeast, 101 are essential for growth in standard laboratory conditions and possess identifiable human orthologs as determined by EggNOG (Huerta-Cepas *et al.* 2016) and InParanoid (Sonnhammer and Östlund 2015) (Figure S1). We focused on orthologs constituting major structural elements of the eukaryotic cytoskeleton, including actin, myosin, septin, and tubulin gene families identifying 106 testable human–yeast ortholog pairs. By restricting our tests to elements of the yeast cytoskeleton essential for cell growth under standard laboratory conditions, we could initially assay replaceability of human genes in yeast via simple growth rescue assays. Since human cytoskeletal gene families have undergone multiple duplication events, we systematically swapped each human gene within a family in place of its corresponding yeast ortholog to assay if present-day human genes could still complement the lethal loss of their yeast ortholog(s).

**Figure 2:**
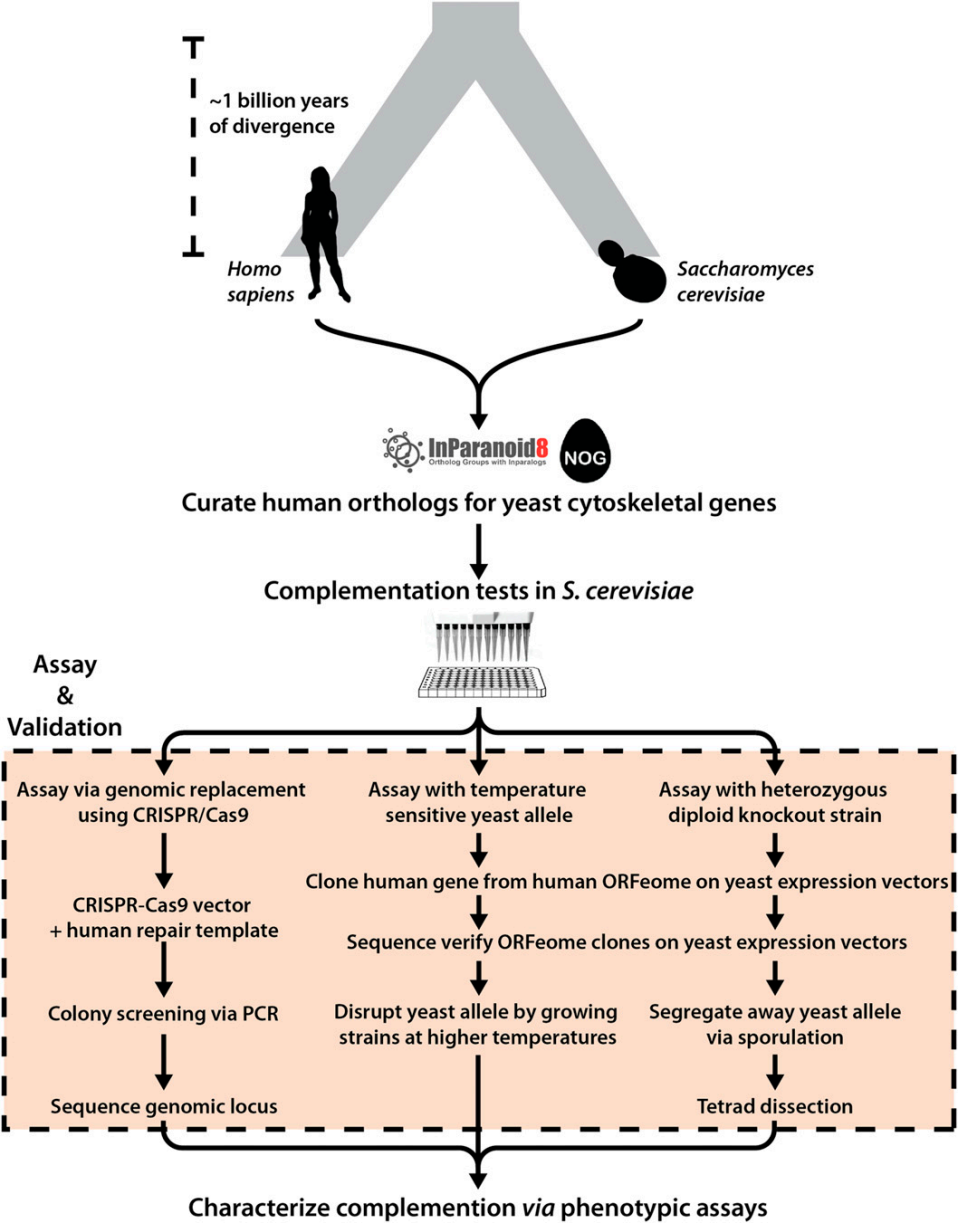
Overview of humanization assays. For each human–yeast ortholog pair (curated from InParanoid and EggNOG), complementation assays in *Saccharomyces cerevisiae* were performed using three strategies: (i) genomic replacement at the native yeast loci via CRISPR-Cas9, (ii) temperature-sensitive inactivation of the yeast allele, and (iii) sporulation of a heterozygous diploid deletion strain followed by tetrad dissection. Complementing human orthologs were further characterized using various phenotypic assays, including quantitative growth measurements, environmental stress tests, mating, and segregation assays.

Complementation assays in yeast were carried out in three different ways (Figure 2), wherein orthologous yeast genes could be (i) genetically segregated away after sporulation of a heterozygous diploid deletion strain (Wach *et al.* 1994; Winzeler *et al.* 1999; Giaever *et al.* 2002) (Figure S2A); (ii) inactivated via a ts allele (Li *et al.* 2011; Kofoed *et al.* 2015) (Figure S3A); or (iii) endogenously replaced by homologous recombination repair with the human ortholog(s) following CRISPR-Cas9 cleavage (Kachroo *et al.* 2017; Akhmetov *et al.* 2018) (Figure S4A).

**Figure 3:**
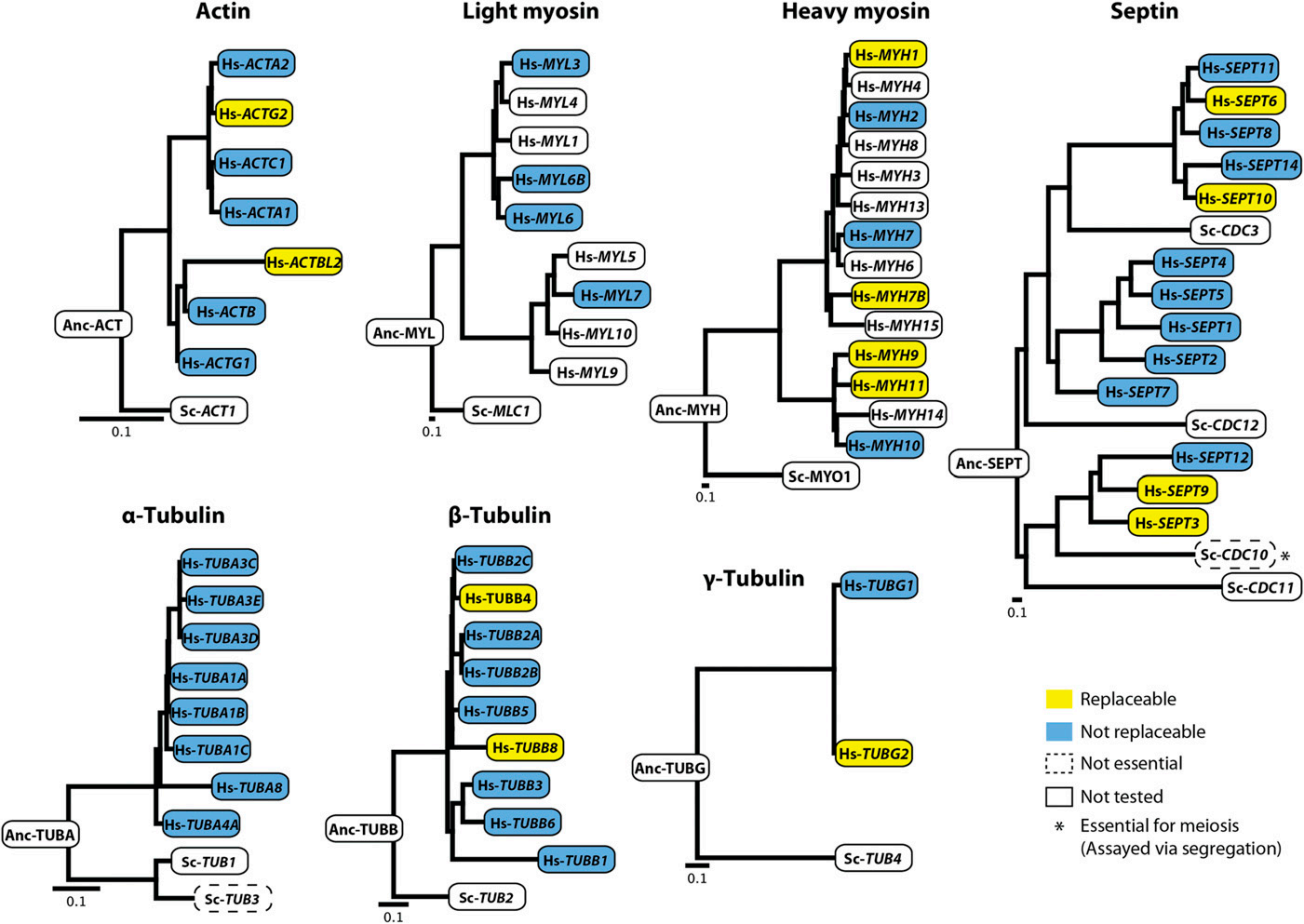
Human cytoskeletal genes replace their corresponding yeast orthologs. Five of seven tested cytoskeletal families possess at least one functionally replaceable human ortholog. None of the tested human light myosin or α-tubulin genes could replace their corresponding yeast versions. Each human septin ortholog was tested for replaceability in four yeast septin null backgrounds (*CDC3*, *CDC10*, *CDC11*, *CDC12*); however, human septins complemented only *CDC10*, * nonessential for mitotic growth but essential in segregation and mating. (Orthology relationships between human genes and yeast septins *SHS1*, *SPR3*, *SPR28* not shown). All phylogenetic gene trees were constructed with protein sequences of their respective orthologs (see *Materials and Methods*). Scale bars indicate expected substitutions per site.

Each assayed human gene was sequence verified and either subcloned into a single-copy (CEN) yeast expression vector transcriptionally controlled by a constitutive GPD promoter [in assays (i) and (ii)] or PCR amplified with flanking homology to the yeast locus of interest [in assay (iii)]. In cases involving heterozygous diploid sporulation assays, we leveraged the heterozygous diploid deletion collection (Giaever *et al.* 2002), wherein one copy of the yeast ortholog being queried has been replaced with a KanMX resistance module, thus enabling selection of haploids with either the null (conferring G418 antibiotic resistance) or the wild-type allele (susceptible to G418) post sporulation (Figure S2, B and C). In cases of ts haploid strains, a single gene of interest is modified to carry mutations inactivating its corresponding gene product at restrictive temperatures (37°) but permitting growth at permissive temperatures (25°), thus allowing for temperature-dependent growth rescue complementation assays (Figure S3, B and C). Finally, in cases of endogenously chromosomal replacement, human genes were assayed by natively substituting their yeast ORF mediated via CRISPR-Cas9 (Akhmetov *et al.* 2018) (Figure S4, B and C).By taking advantage of full-length human cDNA clones from the human ORFeome (Rual *et al.* 2005; Lamesch *et al.* 2007; MGC Project Team *et al.* 2009) and existing yeast strains with null (or conditionally null) alleles for the relevant orthologs (Winzeler *et al.* 1999; Giaever *et al.* 2002; Li *et al.* 2011; Kofoed *et al.* 2015), we successfully assayed 89 of 109 human–yeast ortholog pairs, accounting for 50 of 62 (∼81%) human genes across the seven major cytoskeletal gene families. We observed that 13 (∼26%) of the 50 tested human genes could functionally replace their yeast orthologs in at least one of the three assay types, while 37 (∼72%) could not (Figure 2 and Table S1 and S2). In particular, we found that 2 of 7 actin, 4 of 14 myosin (heavy chain), 4 of 13 septin, 2 of 9 β- and 1 of 2 γ-tubulin human genes complemented lethal growth defects caused by loss of their corresponding yeast orthologs, whereas genes from the human light chain myosin and α-tubulin families did not (Figure 3). Since the septin family had expansions in both human and yeast lineages, we systematically assayed all human septins against four yeast deletion backgrounds (*CDC3*, *CDC10*, *CDC11*, and *CDC12*), observing that complementing human septins functionally replaced only *CDC10*. Thus, within five of these seven essential cytoskeletal gene families, at least one extant human gene could substitute for its yeast ortholog, indicating that the yeast and human genes both still executed the essential roles of their shared opisthokont ancestor.

To assay robustness of complementation and better measure the extent to which human orthologs complemented in standard laboratory conditions, we performed quantitative growth assays on the humanized strains. We measured growth at 30° (where possible, ts strains were measured at 37°) (Figure S5). Humanized strains assayed via plasmid based complementation of the heterozygous diploid sporulation assay were assayed both in selective (SC-Ura) and rich (YPD) medium. While retaining the human-gene-encoding plasmid, these strains showed no major growth defects in YPD. However, they exhibited significantly slower growth rates in SC-Ura medium compared to their wild-type yeast counterparts (Figure S5A). Besides Hs-*ACTBL2* and Hs-*SEPT3*, all other humanized strains showed drastically reduced growth rates in SC-Ura selective medium [Figure S5A(i)] compared to wild-type yeast. In particular, we found that strains with humanized heavy myosins and septins had mean doubling times over twice as long as wild-type [Figure S5A(i) and Table S3]. However, in rich medium (YPD), these fitness defects were substantially rescued [Figure S5A(ii)]. Particularly, yeast strains with Hs-*MYH9* affected growth to saturation phase in YPD, with doubling times twice that of the wild-type rate [Figure S5A (ii) and Table S3]. In the case of ts growth rescue assays at 37°, we found that humanized strains assayed generally exhibited slower growth rates. In particular, humanized γ-tubulin (Hs-*TUBG2*) strains exhibited drastically slow growth rates, doubling at one-quarter the rate of wild-type strains and failing to reach comparable biomass at saturation (Figure S5B and Table S3). In contrast, the genomically replaced β-tubulins Hs-*TUBB4* and *Hs-TUBB8* both shared similar growth profiles to wild-type yeast strains (Figure S5C and Table S3).

To probe growth effects of humanization in more detail, we subjected the strains (except those assays performed in a ts strain background) to temperature stress, repeating the growth assays at low (25°) and high (37°) temperatures. While most humanized strains grew similar to wild-type rates at 25° (Figure S11 bottom), we observed differential effects at 37° (Figure S11 top). Specifically, yeast with human septins Hs-*SEPT6*, Hs-*SEPT9*, and Hs-*SEPT10* all reached stationary phase at a lower biomass compared to the wild-type yeast. Although septin genes complemented the lethal growth defect, they performed suboptimally, especially under stress conditions, suggesting that they may be failing to complement other critical roles of the yeast orthologs, meriting a deeper examination of additional gene family specific phenotypes, especially in light of recent observations that *cdc10**Δ* phenotype severity may also be modulated by altering plasma membrane properties (Michel *et al.* 2017).

### Sporulation and meiotic roles of human β-tubulin are regulated by their native regulation in yeast

**Figure 4:**
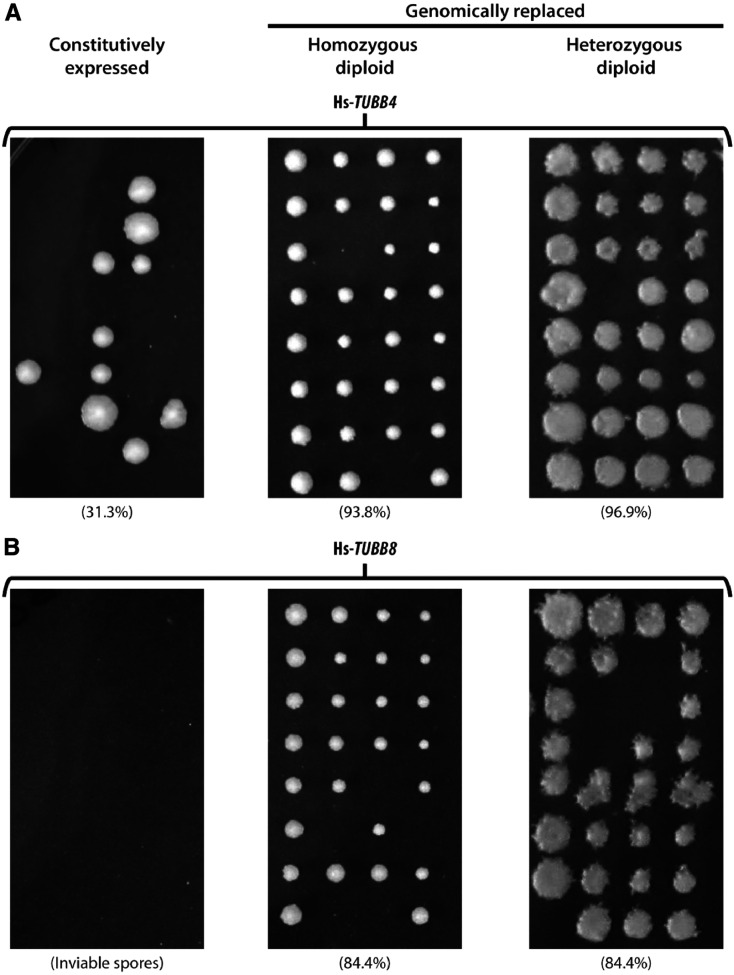
Replaceability of human β-tubulins Hs-*TUBB4* and Hs-*TUBB8* is determined by their native yeast expression and regulation. Efficiency of sporulation and segregation of both (A) Hs-*TUBB4* and (B) Hs-*TUBB8* tested in three different genetic backgrounds *via* tetrad dissection. Both constitutively expressed human β-tubulins Hs-*TUBB4* and Hs-*TUBB8* (controlled by a GPD promoter) fail to complement (left panel) whereas when replaced genomically could both grow and mate with wild-type yeast (middle panel). Diploids (both hetero- and homozygous) could also proceed through sporulation and meiosis similar to the wild-type yeast diploid strain (middle and right panel, see *Materials and Methods*). For population-level complementation assays of the heterozygous diploid deletion strains see Figure S6. Spore viability indicated in brackets.

In yeast, β-tubulin (*TUB2*) is primarily associated with chromosome segregation during budding (Neff *et al.* 1983; Vogel *et al.* 2001; Bode *et al.* 2003) (asexual reproduction involving mitosis), mating (Bardwell 2005; Molk and Bloom 2006) (sexual reproduction) and sporulation (Neiman 2011) (starvation response involving meiosis). In our complementation assays, we observed that the human β-tubulins Hs-*TUBB4* and Hs-*TUBB8* only complemented the loss of *TUB2* when genomically inserted at the yeast *TUB2* locus but failed to do so in plasmid-based segregation assays in heterozygous diploid knockout strains. Notably, plasmid expression was controlled transcriptionally by a constitutive GPD promoter; both human genes failed to rescue its corresponding heterozygous diploid yeast gene deletion leading to sporulation defects producing either mis-segregated (Figure 4A, left panel) or inviable spores (Figure 4B, left panel). Therefore, replacing endogenous *TUB2* with human β-tubulins Hs-*TUBB4* and Hs-*TUBB8* at a minimum supported mitosis and asexual cell division via budding, but it was unclear if they supported meiosis and sporulation.

In order to determine whether replaceable human β-tubulins also rescued meiotic and sporulation specific roles of their yeast ortholog, we mated haploid humanized strains (harboring human genes at the corresponding yeast genes’ native genomic loci) with wild-type yeast. These humanized strains successfully mated with wild-type yeast strains to produce viable diploids. We assayed if these heterozygous humanized strains could successfully perform meiosis by sporulating the diploids generated from our mating assays. We observed high spore viability for both Hs-*TUBB4* (∼96.9%) and Hs-*TUBB8* (∼84.4%) (Figure 4, A and B, right panel). Having shown that both replaceable β-tubulins can mate with the wild-type strains (yielding high sporulation efficiency similar to the wild-type diploids) (Figure S6A, right panel), we next asked whether strains homozygous for human β-tubulins could behave similarly. We sporulated the humanized homozygous β-tubulin diploid strains assaying for proper progression through meiosis via sporulation, and found that these diploid humanized yeast strains also sporulated in both Hs-*TUBB4* (93.8%) and Hs-*TUBB8* (84.4%) cases. (Figure 4, A and B, middle panel) similar to their heterozygous (Figure 4, A and B, right panels) and wild-type diploids (∼87.5%) (Figure S6A, right panel including 2:2 segregation of the MAT, LYS and MET loci after dissecting tetrads, see Figure S6). These results reveal that native regulation of β-tubulin is more critical to yeast than the species origin of the gene. The functional replacement of *TUB2* by two human orthologs, Hs-*TUBB4* and Hs-*TUBB8*, requires insertion into the native yeast locus, but nonetheless enables these genes to successfully perform their roles in meiosis and sporulation, similar to the yeast β-tubulin *TUB2* (Figure S6**)**

### Human septin orthologs and their isoforms can carry out the meiotic and mating role of *CDC10*

Next, we considered if human orthologs of yeast septins could also perform conditionally essential cytoskeletal roles in yeast. The yeast septin family is a particularly interesting case of gene family expansion as there have been duplications in both the human and yeast lineages, suggesting that ancestral functions might have been distributed across paralogs in both species. Yeast possess seven septin genes (*CDC3*, *CDC10*, *CDC11*, *CDC12*, *SPR3*, *SPR28*, and *SHS1*) while humans have 13 septin genes (Pan *et al.* 2007) (Figures 1 and 3). Three of the seven yeast septins (*CDC3*, *CDC11*, and *CDC12*) are essential for vegetative growth (budding) in standard laboratory conditions. We performed 39 of the 91 possible septin complementation assays in each of the three septin null backgrounds, and found that none of the human orthologs assayed could complement *CDC3*, *CDC11*, and *CDC12*.

While the remaining four septins (*CDC10*, *SPR3*, *SPR28*, and *SHS1*) are not essential for vegetative growth, *CDC10* plays essential roles in meiotic cell division, mating, and spore health, with *cdc10**Δ* strains exhibiting severe mating and sporulation defects (Douglas *et al.* 2005; McMurray *et al.* 2011; Neiman 2011; Kim and Rose 2015). Indeed, we also observed that the diploid strain heterozygously null for *CDC10* sporulated poorly, almost always producing only two viable spores [Figure S2B(ii)].

**Figure 5:**
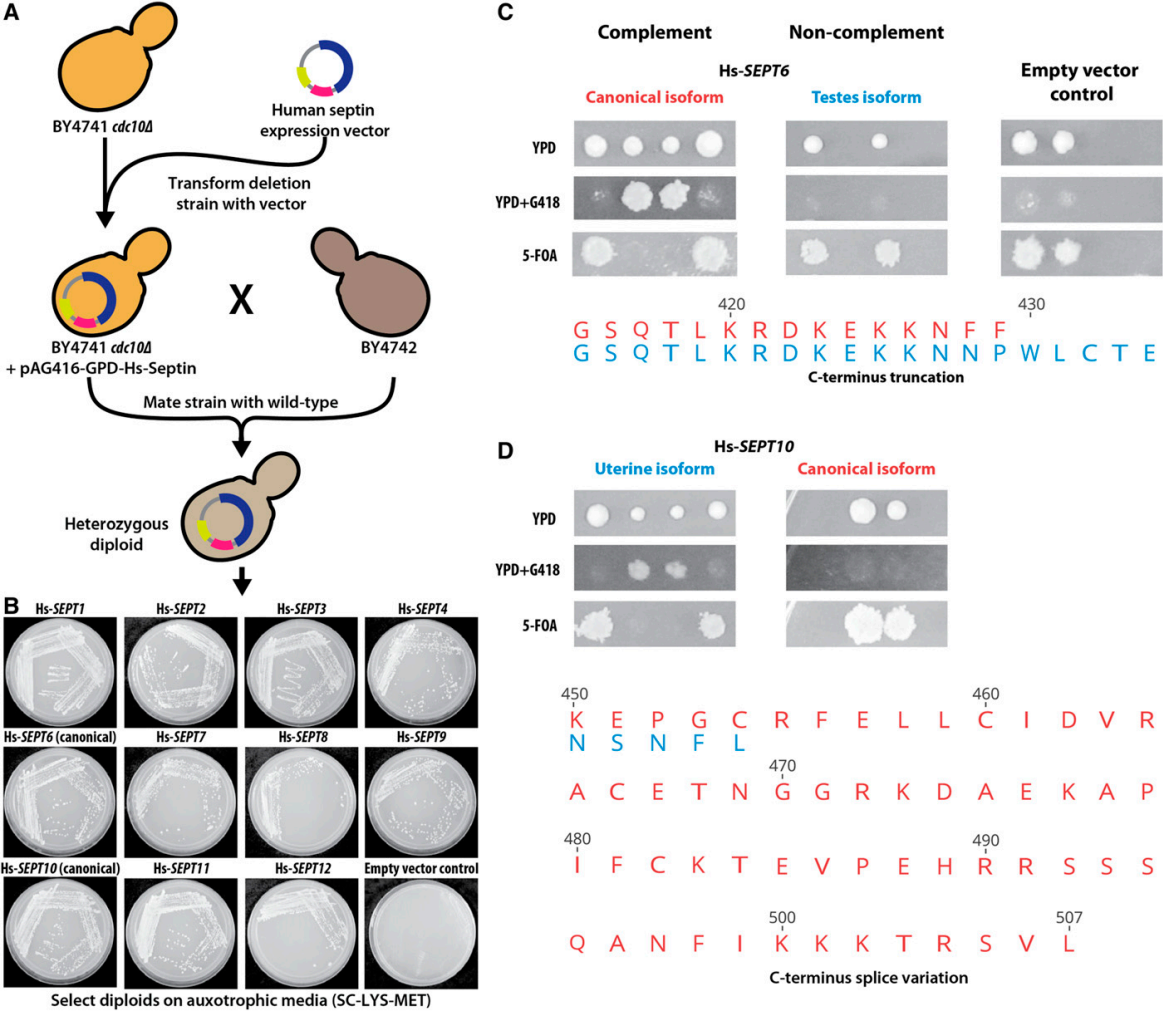
Human septins differentially rescue meiotic and mating roles of the yeast *CDC10*. (A) Mating rescue assay for BY4741 *cdc10**Δ*. (B) All assayed human septins expressing strains can mate with BY4742, whereas the empty vector containing BY4741 *cdc10**Δ* fails to rescue the mating defect caused by deleting *CDC10*. Plates were imaged after 3 days of incubation on SC-Lys-Met, (C) and (D) depict differential replaceability of human septin splice forms, Hs-*SEPT6* and Hs-*SEPT10*, respectively. The top panels demonstrate the heterozygous diploid deletion mutant segregation assay for the assayed isoforms while the bottom panels show the sequence alignment of the variation across the canonical (red) and tissue-specific (blue) isoforms tested for both Hs-*SEPT6* and Hs-*SEPT10*.

To identify human orthologs capable of rescuing sporulation defects caused by the loss of *CDC10*, we systematically assayed each of the 13 human septin orthologs. We observed that four of 13 septins (Hs-*SEPT3*, Hs-*SEPT6*, Hs-*SEPT9*, Hs-*SEPT10*) were capable of functionally complementing the meiotic and sporulation roles of *CDC10* (Figure 3). Since *cdc10**Δ* strains additionally show severe mating defects (McMurray and Thorner 2008; McMurray *et al.* 2011) (Figure S7A), we next asked if the expression of individual human septin orthologs in a *cdc10**Δ* strain could facilitate mating. We assayed this by cloning human septin genes into yeast expression vectors (*Materials and Methods*) and individually transformed *cdc10**Δ* strains with each human septin clone. Subsequently, we mated each *MATa* transformant to a complementary *MATα* strain (Figure 5A). While only four of 13 tested human septins, Hs-*SEPT3*, Hs-*SEPT6*, Hs-*SEPT9*, and Hs-*SEPT10*, rescued the essential meiotic role of *CDC10*, we found that all assayed septins were capable of performing *CDC10*’s role in mating (Figure 5B).

Unlike in yeast, human septins are known to exhibit a variety of different splice forms (Hall and Russell 2004; Weems and McMurray 2017). Based on the availability of verified splice forms (as per the Uniprot database) in the human ORFeome, we tested different isoforms of Hs-*SEPT6* and Hs-*SEPT10*, observing contrasting results with both sets of isoforms. While the canonical isoform of Hs-*SEPT6* functionally complemented its yeast ortholog *CDC10*, its testes isoform with an extended C-terminus failed to do so (Figure 5C). However, when testing the uterine and canonical isoforms of Hs-*SEPT10*, the opposite was true, with only the truncated uterine form functionally complementing *CDC10* (Figure 5D). Thus, the presence of an extended C-terminus negatively affected the ability of the tested human septin isoforms to replace their yeast ortholog. Taken together, these results suggest that human septin orthologs can effectively rescue essential *CDC10* phenotypes in mating and meiosis.

### Humanized yeast strains differ in cell morphology

**Figure 6:**
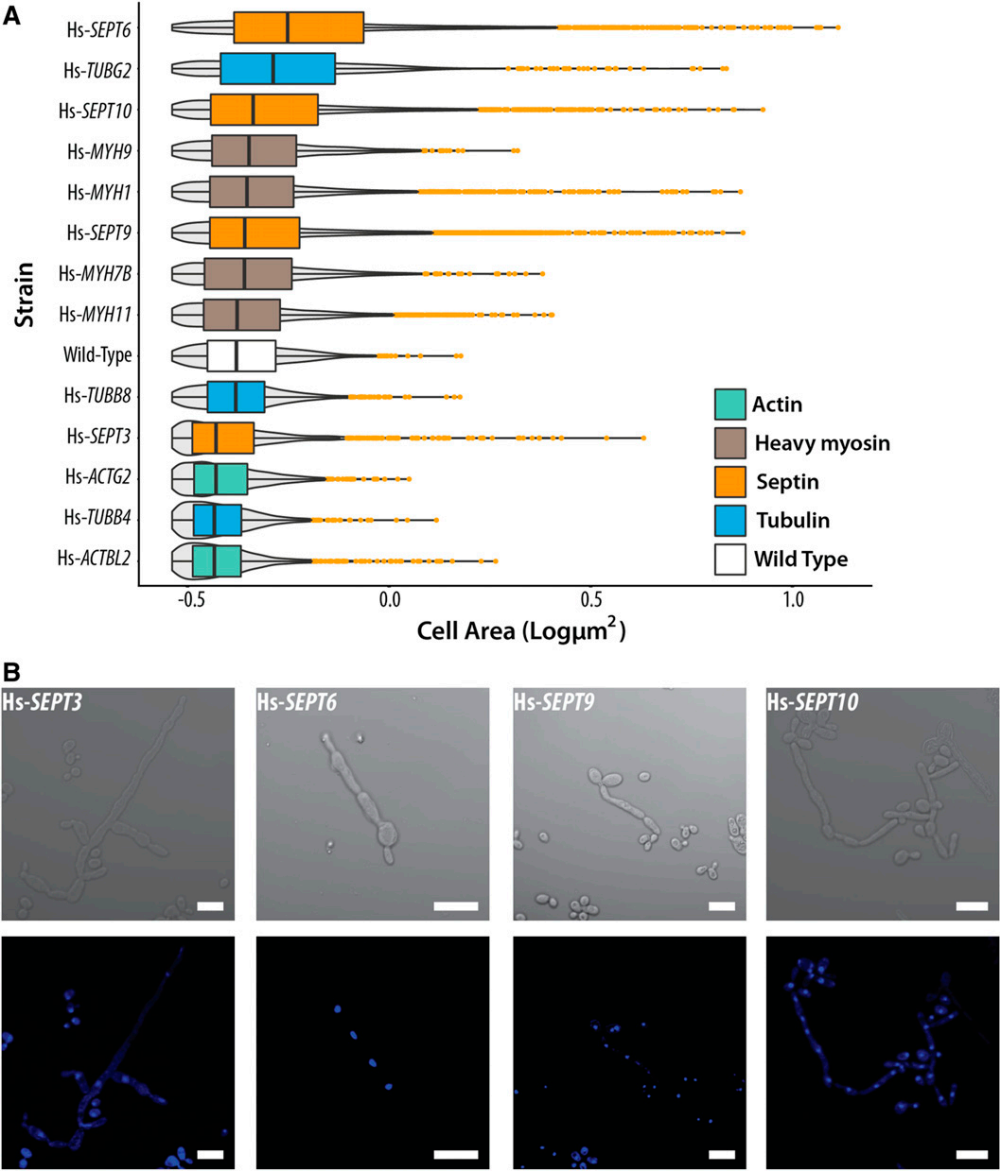
Yeast strains with humanized cytoskeletal components exhibit distinct cellular morphologies. (A) Humanized strains show varying cell sizes. Cell areas of humanized strains (in square pixels) are plotted on the *X*-axis. Gray violins indicate cell size distributions. Orange dots indicate outliers. Humanized actin strains show reduced cell size while myosins and tubulins (except γ-tubulin) largely remain unchanged. However, humanized septin strains show drastically elongated cellular morphologies. Significance comparisons with wild type determined by standard *t*-test with ***P ≤ 0.001; **0.001 < *P* ≤ 0.01; *0.01 < *P* ≤ 0.05; NS, *P* > 0.05, not significant. Additional bright-field images are shown in Figure S8. (B) Magnified bright-field and DAPI-stained images of humanized septin strains exhibit elongated morphologies and are multinucleated as a consequence of defective cytokinesis. (Scale bar, 10 µm.)

Eukaryotic cytoskeletal proteins dynamically control cell shape and morphology. We next asked whether humanizing elements of the yeast cytoskeleton would result in any visually obvious phenotypic changes to cell morphology. Using light and fluorescence microscopy, we imaged the 13 humanized strains generated and quantified cell shape and size across >22,000 individual cells (*Materials and Methods*). In the case of humanized heavy myosins, Hs-*MYH7B* and Hs-*MYH11*, we did not observe any obvious defects in cell morphologies, but, introducing Hs-*MYH1* and Hs-*MYH9* induced a slight size increase leading to more spherical cells (Figure 6A and Figure S7). However, the effect, while significant, was small, and their median sizes were within 1% of wild type (Figure 6A and Figure S8). In contrast, strains with humanized actins, tubulins, and septins had visibly different cell morphologies (Figure 6A and Figure S8). Humanizing actin visibly reduced cell size, resulting in round/spherical cells with small buds (Figure 6A and Figure S8). Complementing human γ-tubulin Hs-*TUBG2* showed the opposite phenotype, resulting in enlarged and ovoid cells (Figure 6A and Figure S8). The endogenously replaced human β-tubulin, Hs-*TUBB4*, also showed reduced cell size (Figure 6A and Figure S8). Since α- and β-tubulins physically associate to form heterodimers, we introduced human β-tubulins Hs-*TUBB4* and Hs-*TUBB8* into a strain expressing GFP-tagged α-tubulin (Huh *et al.* 2003) (*TUB1**-GFP*), allowing us to visualize microtubules and observe if chimeric yeast–human αβ-tubulin heterodimers were being assembled (Figure S10A). While we observed microtubules in both strains, we noticed different cell morphologies when humanizing Hs-*TUBB4* (Figure S10A), indicating a synthetic genetic interaction between the GFP tagged form of *TUB1* and Hs-*TUBB4*, absent from the interaction of Hs-*TUBB4* and native *TUB1*. Examining the distributions further, we observed a bimodal distribution of cell sizes (Figure S10B) for Hs-*TUBB4* suggestive of cell cycle defects [as for *TUB2* Ser172 mutations (Caudron *et al.* 2010)]. In contrast, the cell morphology and microtubule assembly of the *TUB1**-GFP* strain humanized with Hs-*TUBB8* appeared similar to wild type.

All replaceable septin strains (Hs-*SEPT3*, Hs-*SEPT6*, Hs-*SEPT9*, and Hs-*SEPT10*) showed abnormal cell morphologies (Figure 6 and Figure S8), notably reminiscent of elongated pseudohyphal forms often observed in septin mutant strains as a function of improper activation of the morphogenesis checkpoint in the budding yeast cell cycle (Keaton and Lew 2006; McMurray *et al.* 2011). In order to verify if the replaceable human septins localized to the bud neck, we expressed C-terminal eGFP-tagged Hs-*SEPT4* (nonreplaceable) and Hs-*SEPT9* (replaceable) in heterozygous diploid deletion strains. First, we observed that when sporulated, the *eGFP*-tagged Hs-*SEPT9* could also rescue the sporulation defect induced by deletion of *CDC10* (Figure S9A), whereas the mating defect in *cdc10**Δ* haploids is rescued by both Hs-*SEPT9* and Hs-*SEPT4 eGFP*-tagged versions (Figure S9B). Second, we tested the localization of both these human septins in haploid *cdc10**Δ* strains. We did not see the localization at the bud neck (Figure S9C); rather, we observed the GFP fluorescence diffused throughout the cell. Therefore, we conclude that the likely mode of the rescue of yeast septin function is partial or indirect in nature. The assay also reveals that the strains do not have any major spore formation defect, as we see several colonies growing in SC-Arg-His-Leu+Can-Ura-G418 medium, similar to the empty vector control transformed strain (Figure S9A). While it has been reported that *cdc10**∆* null mutants do have aneuploid genomes (McMurray *et al.* 2011), in the conditions that we tested (heterozygous diploid deletion sporulation assay in SC-Arg-His-Leu+Can-Ura+/−G418 with growth for 3 days), we rule out aneuploidy as a consequence of haploinsufficiency. However, we also observed that many *cdc10**Δ* haploid colonies that grow in the presence of human gene (Hs-*SEPT9*) were able to also grow independent of the plasmid, suggesting that the replaceability is not optimal, and the expression of Hs-*SEPT9* induces segregation defects in these cells that may likely induce aneuploidy (Figure S9A).

Taken together, these data suggest that human septins fail to fully complement the role of *CDC10*. The fraction of elongated cells differed across human septins, with Hs-*SEPT3* producing lower proportions of elongated cells (Figure 6A and Figure S8). To assay whether this effect arose from defective cytokinesis, we quantified the nuclei per cell by DAPI staining. We indeed observed that the cells were multinucleated (Figure 6B). Despite this severe cell morphology defect, all replaceable human septins still enabled the strains to maintain growth rates comparable to wild-type strains (Figure 3D). Taken together, we found that multiple human septins can rescue the essential meiotic and segregation roles of *CDC10*, but result in abnormal cell morphologies with delayed and/or defective cytokinesis.

### Humanized cytoskeletal orthologs phenocopy cell morphology defects observed by deleting interaction partners

**Figure 7:**
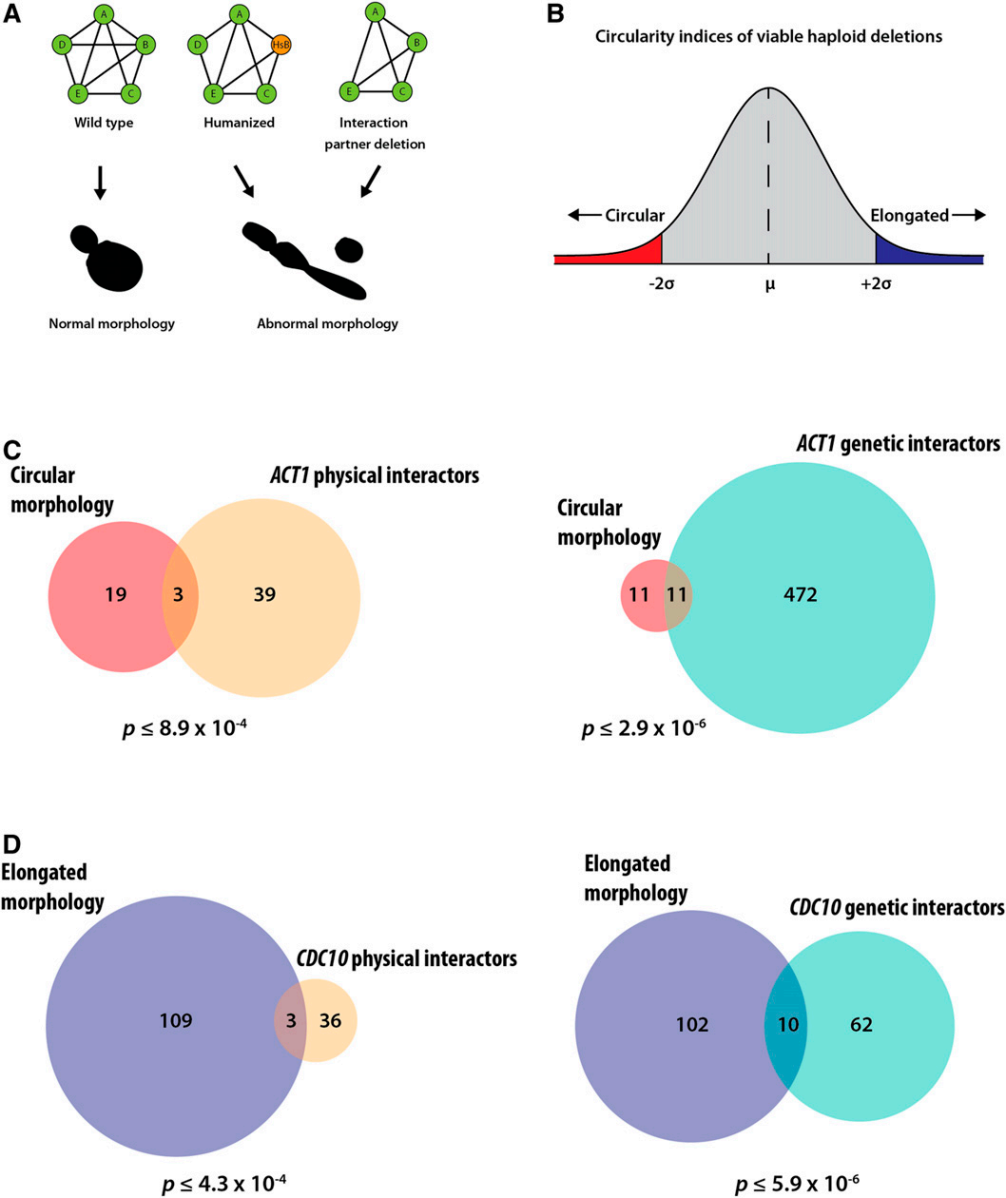
Humanized cytoskeletal yeast strains with abnormal morphologies phenocopy deletions of their corresponding yeast or tholog’s interaction partner. (A) Schematic depicting the nature of physical/genetic interactions in wild-type (Left), humanized (Middle), and interacting partner deletion (Right) yeast strains. Humanized yeast and the deletion strains are shown to cause similar abnormal cell shapes. (B) Distribution of circularity indices of viable haploid deletion strains. Gene deletions leading to significantly elongated and circular morphologies would lie in the blue and red regions respectively. (C) Overlaps of gene deletions leading to circular morphology with *ACT1* physical (left) and genetic (right) interactors. (D) Overlaps of gene deletions leading to elongated morphologies with *CDC10* physical (left) and genetic (right) interactors. Cell shape parameters computed from data from SCMD. *ACT1* and *CDC10* interactors curated from SGD (for additional details see Table S5). P-values determined by hypergeometric test.

In spite of rescuing lethal growth defects, the humanized strains showed visible cell morphology defects consistent with the known roles of the humanized genes. These defects suggested that the complementing human genes might be failing to perform some of the nonessential roles of the yeast cytoskeletal orthologs regulating cell shape and morphology. While the replaceable human cytoskeletal genes functionally complement the lethal growth defect caused by deletion of their yeast orthologs, we hypothesized that they failed to fully interface with their constituent interaction networks, thereby breaking key nonessential interactions regulating cell morphology. If true, this would imply that the humanization of a particular cytoskeletal yeast gene might phenocopy the deletion of its corresponding nonessential yeast interaction partner(s) (Figure 7A).

To test this hypothesis, we curated both physical and genetic interaction partners of all humanizable yeast orthologs from the SGD (Cherry *et al.* 1998, 2012) and mined the SCMD (Saito *et al.* 2004), a database cataloging the morphologies of ∼1.9 million cells across 4718 haploid nonessential gene deletion backgrounds in yeast and measuring ∼501 cell shape parameters. Specifically, we considered the humanized strains showing drastic morphology changes, and computed the ratio of long to short cell axes as a measure of cell roundness or elongation. Deletion strains with elongated phenotypes (*i.e.*, similar to humanized septin strains) would have higher axes ratios, and strains with round/spherical cells (*i.e.*, similar to humanized actin strains) would have axes ratios centered around 1. To ensure stringency in curating interactors of both *ACT1* and *CDC10*, we restricted the interactions *a priori* to those reported at least twice in the literature. Additionally, we further restricted the interacting partners list to only those that contained SCMD cell morphology measurements for the corresponding deletion strain (Table S5). To test our hypothesis, we next plotted the z-scores of the circularity index, and asked whether there was a significant enrichment of *ACT1* interactors in deletion strains with circular morphologies (<2 standard deviations) and contrastingly an enrichment of *CDC10* interactors in deletion strains with elongated morphologies (>2 standard deviations) (Figure 7B). Indeed, we found a significant overlap, albeit not a very large one, between both the physical and genetic interactors of humanizable cytoskeletal genes and their observed phenotypes when deleted. In particular we found 3 physical (*P* ≤ 8.9 × 10^−4^) and 11 genetic interactors (*P* ≤ 2.9 × 10^−6^) of *ACT1* enriched for circular morphologies. In the case of yeast septins, we found 3 physical (*P* ≤ 4.3 × 10^−4^) and 10 genetic (*P* ≤ 5.9 × 10^−6^) interactors of *CDC10* enriched for elongated morphologies (Figure 7, B and C). Taken together, these data suggest that the replaceable human orthologs may indeed fail to maintain nonessential physical interactions controlling cell shape and morphology and genetically perturb relevant morphology genes. It is possible that the amplification of cytoskeletal elements in the human lineage may have led to distribution of specific subfunctions and/or loss of interactions across duplicated human genes compared to the opisthokont ancestor. Our observations suggest that replaceable human cytoskeletal genes functionally complement the essential roles of their yeast ortholog, but simultaneously break key nonessential cytoskeletal associations, thereby phenocopying the deletion of their interaction partners.

## Discussion

Identifying how genes retain and/or distribute roles across their families is key to understanding the diversification of conserved genes across organisms. Cross-species gene swaps provide an opportunity to directly test the functional divergence of orthologous genes even over large evolutionary timescales. While *S. cerevisiae* (Baker’s yeast) and *Homo sapiens* (humans) diverged from their opisthokont ancestor nearly a billion years ago, both species still share thousands of orthologous genes, and high-throughput humanization assays in yeast have found that many human genes are capable of substituting for their yeast orthologs with rates up to 47%, depending on strains and assays (Kachroo *et al.* 2015; Hamza *et al.* 2015; Laurent *et al.* 2016, 2019; Sun *et al.* 2016; Yang *et al.* 2017). Most of these complementation tests until recently were performed in the absence of gene family expansions and revealed many humanizable systems, including the proteasome, sterol and heme biosynthesis pathways (Kachroo *et al.* 2015, 2017; Hamza *et al.* 2015; Sun *et al.* 2016; Yang *et al.* 2017; Laurent *et al.* 2019). In this study, we sought to better understand the functional equivalence of human and yeast orthologs that play key structural roles in the eukaryotic cytoskeleton, particularly focusing on how gene family expansions in cytoskeletal lineages might have diversified in function across their respective gene families.

By systematically humanizing key structural components central to the yeast cytoskeleton, we determined that five of the seven (∼71%) assayed eukaryotic cytoskeletal gene families could be successfully humanized by at least one human ortholog within a family. In all, 13 (26%) out of 50 tested human cytoskeletal proteins could, at least partially, substitute for the corresponding yeast gene and complement a lethal growth defect caused upon loss of the yeast ortholog (Figure 3A). Interestingly, these results are broadly consistent with our previous study, where 17 (∼28%) of 60 tested regulatory genes of the human cytoskeleton replaced their yeast orthologs (Laurent *et al.* 2019) (Figure S1).

It is noteworthy that we were unable to successfully humanize yeast with any member of the myosin light chain or α- tubulin gene families, suggesting extensive functional divergence in yeast–human ortholog pairs resulting in failure to substitute for each other across species. This variation in replaceability was not merely explainable by obvious patterns across human genes (at least as captured by the variation in their expression patterns across tissues; Figure S12A), nor was it explained by their degree of sequence similarity. As with previous yeast humanization studies (Kachroo *et al.* 2015), sequence conservation among replaceable and nonreplaceable human cytoskeletal genes did not significantly predict replaceability (Figure S12B). While uncovering additional new properties predicting replaceability is beyond the scope of this study, future efforts at systematically constructing chimeric human/yeast genes have the potential to reveal which regions of the human/yeast orthologs are critical to maintain functional compatibility, perhaps enabling targeted humanization of specific domains and regions of genes (Barger *et al.* 2019).

With only slight differences in mitotic growth between wild-type and most humanized strains observed under standard laboratory growth conditions, our results suggest robust complementation of essential cellular roles in most cases. However, we subsequently found incomplete complementation of multiple nonessential cellular roles. We found that human β-tubulins Hs-*TUBB4* and Hs-*TUBB8* remarkably complemented the roles of *TUB2* even in sexual reproduction, including sporulation and mating. However, this was contingent on genomic integration under the native yeast regulation, consistent with previous studies showing that *TUB2* expression levels are tightly regulated (Beilharz *et al.* 2017) with overexpression of β-tubulin leading to toxicity, chromosome loss, and cell cycle arrest (Burke *et al.* 1989; Weinstein and Solomon 1990; Abruzzi *et al.* 2002).

For the septin gene family, which has expanded to consist of seven yeast genes to 13 in humans, we found evidence for functional divergence across family members in both human and yeast lineages, with human genes tending to only fulfill a subset of specific roles of their yeast ortholog(s). While none of the human septin orthologs individually rescued essential roles of the yeast *CDC3*, *CDC11*, and *CDC12* genes, we found that four human septin orthologs (Hs-*SEPT3*, Hs-*SEPT6*, Hs-*SEPT9*, Hs-*SEPT10*) complemented the essential roles of *CDC10* in meiosis and sporulation. However, severe mating defects caused by deleting *CDC10* in a haploid strain background were completely rescued by every assayed member of the human septin family (Figure 5, A and B), indicating that all human septin orthologs executed the roles of their yeast counterparts to at least some extent. Extending this theme further, we saw that replaceability of Hs-*SEPT6* and Hs-*SEPT10* differed based on their protein isoforms, demonstrating functional divergence not just across human septin paralogs but also within splice forms of a gene (Figure 5, C and D). Taken together, such specific complementation patterns suggest a complex evolutionary trajectory and delegation of function across the septin gene family in eukaryotes. It remains to be seen if human septin orthologs can individually complement their nonessential yeast counterparts *SHS1*, *SPR3*, and *SPR28*. While septin gene family expansions have been understood to predominantly bring functional redundancy and robustness within its interactome, recent studies have identified tissue-specific roles within human septin orthologs (Fujishima *et al.* 2007; Tsang *et al.* 2008; Kaplan *et al.* 2017). The mechanical roles facilitated by human and yeast septin gene families appear to be remarkably conserved despite their involvement in seemingly unrelated processes across species (Falk *et al.* 2019).

Over the course of these complementation assays, we observed diverse cell morphologies among humanized strains that were broadly gene family specific, with complementing myosins and β-tubulins largely remaining unchanged, but actin, γ-tubulin, septin families showing characteristic morphological differences as drastic as defective cytokinesis. Owing to the crucial role of the cytoskeleton in maintaining cell shape, we found that replaceable human cytoskeletal genes tend to perform its yeast ortholog’s essential roles equivalently while simultaneously breaking key nonessential cytoskeletal genetic interactions regulating cell morphology. In agreement with our findings, a recent study systematically determining cell size regulators in yeast found that 145 genes of ∼400 deletion strains were genetic interactors of actin (Soifer and Barkai 2014). Replaceable human septin orthologs, in particular, represent interesting and rather extreme cases of abnormal cell morphology. Previously, a study showed that introducing the *Aspergillus nidulans* septin *AspC* in *S. cerevisiae* induces a similar pseudohyphal morphology observed when substituting for *CDC12* in the septin ring (Lindsey *et al.* 2010). More recently, a report (Kim and Rose 2015) demonstrated that doubly deleting *ELM1* and *FUS3* (both genetic interactors of *CDC10*) in yeast produces the same filamentous elongated morphology with similar cytokinesis defects observed when humanizing *CDC10*. In agreement with these studies, our results suggest that human septins sufficiently complement the roles of *CDC10* by surpassing cellular thresholds for growth but not morphology. While in cases of both yeast actin and septin, we indeed find significant enrichment of their corresponding genetic interactors leading to abnormal morphologies, it is difficult to mechanistically interpret the consequences of genetic interactions or lack thereof. However, with significant enrichment in the physical interactors of *ACT1* and *CDC10* associated with the observed cell morphologies, our analyses suggest that human cytoskeletal orthologs in yeast may break at least some nonessential protein–protein interactions underlying normal cell morphology. It remains to be seen if humanization of their corresponding interaction partners can revert yeast cells to normal wild-type like morphologies. The genetic and biochemical mechanisms by which human orthologs regulate cytoskeletal interactions in yeast are yet to be explored.

Systematic swaps of humanized cytoskeletal elements in yeast now provide a direct view of how compatible human orthologs likely are within their corresponding yeast interaction network(s), pointing to conserved and divergent interactions among eukaryotes. While our complementation assays tested the ability of human cytoskeletal orthologs to singly complement their yeast equivalents, combinatorial multi-gene swaps might enable humanization of entire yeast systems to study modularity and paralog-level cross-talk between different human cytoskeletal families in a genetically tractable eukaryote. Widening the scope of cytoskeletal humanization efforts to include accessory motors and chaperones, including kinesins and dyneins, could help advance our understanding of eukaryotic cytoskeletal evolution. It remains to be seen if constructing a fully human cytoskeleton in yeast would be feasible.

These humanized strains can now serve as cellular reagents to study complex human cytoskeletal processes in a simplified eukaryotic context, allowing functional roles of distinct family members to be assayed individually. Screening allelic variants and mutational libraries using these strains might enable the rapid identification of disease variants in a high-throughput manner (Kachroo *et al.* 2015; Hamza *et al.* 2015; Sun *et al.* 2016, 2018; Yang *et al.* 2017; Marzo *et al.* 2019), paving the way for a better understanding of the genetic and molecular basis of cytoskeletal disorders.
